# Histoplasmosis in Africa: An emerging or a neglected disease?

**DOI:** 10.1371/journal.pntd.0006046

**Published:** 2018-01-18

**Authors:** Rita O. Oladele, Olusola O. Ayanlowo, Malcolm D. Richardson, David W. Denning

**Affiliations:** 1 Department of Medical Microbiology and Parasitology, Faculty of Basic Medical Sciences, College of Medicine, University of Lagos, Lagos, Nigeria; 2 Faculty of Biology, Medicine and Health, The University of Manchester, Manchester Academic Health Science Centre, Manchester, United Kingdom; 3 Department of Medicine, Faculty of Clinical Sciences, College of Medicine, University of Lagos, Lagos, Nigeria; 4 Mycology Reference Centre Manchester, Wythenshawe Hospital, Manchester University NHS Foundation Trust, Manchester, United Kingdom; 5 National Aspergillosis Centre, Wythenshawe Hospital, Manchester University NHS Foundation Trust, Manchester, United Kingdom; 6 Global Action Fund for Fungal Infections, Geneva, Switzerland; University of California San Diego School of Medicine, UNITED STATES

## Abstract

Histoplasmosis in Africa has markedly increased since the advent of the HIV/AIDS epidemic but is under-recognised. Pulmonary histoplasmosis may be misdiagnosed as tuberculosis (TB). In the last six decades (1952–2017), 470 cases of histoplasmosis have been reported. HIV-infected patients accounted for 38% (178) of the cases. West Africa had the highest number of recorded cases with 179; the majority (162 cases) were caused by *Histoplasma capsulatum* var. *dubuosii* (Hcd). From the Southern African region, 150 cases have been reported, and the majority (119) were caused by *H*. *capsulatum* var. *capsulatum* (Hcc). There have been 12 histoplasmin skin test surveys with rates of 0% to 35% positivity. Most cases of Hcd presented as localised lesions in immunocompetent persons; however, it was disseminated in AIDS patients. Rapid diagnosis of histoplasmosis in Africa is only currently possible using microscopy; antigen testing and PCR are not available in most of Africa. Treatment requires amphotericin B and itraconazole, both of which are not licensed or available in several parts of Africa.

## Introduction

Inhalation of conidia of *H*. *capsulatum* leads to histoplasmosis in some people. Hcc is patchily distributed around the world, whereas Hcd is essentially restricted to Africa [1]. Histoplasmosis was first described by Darling in the Canal Zone in Panama in 1906; patients were described as presenting with features suggestive of disseminated TB [2]. The first case of Hcd was described in West Africa in 1943 by Duncan [3].

The true global burden of histoplasmosis is not well documented despite its endemicity and not addressed previously for Africa. Histoplasmosis is not a notifiable disease, thus hard data on the incidence and prevalence, as well as information on its morbidity and mortality, are fragmentary or not available in many endemic areas [4]. Recently, WHO broadened their list of core neglected tropical diseases (NTDs) to include deep mycoses, of which histoplasmosis is one [5]. The greatest attributable risk factor for histoplasmosis is the spread of HIV, although immunosuppressive agents used in transplant patients or chronic inflammatory diseases also contribute to its increase [6]. Disseminated histoplasmosis was classified as an AIDS-defining infection in 1987 [7].

During highly active antiretroviral therapy (HAART), morbidity and mortality due to histoplasmosis remain a public health problem in low- and middle-income countries (LMICs) [4]. Primary infection is extremely common in highly endemic areas based on the prevalence of skin test reactivity, with 23% to 81% and 5% to 50% of the population testing positive in Guatemala and Mexico, respectively [8,9]. In these areas, progressive disseminated histoplasmosis (PDH) can occur in 5% to 20% of patients infected with HIV [1,10]. However, in immunocompetent persons, it is mostly asymptomatic or spontaneously self-limiting [11].

Histoplasmosis is highly prevalent in areas along the Mississippi and Ohio valleys in the United States and in Central and South America [10]. It is also endemic in India and Southeast Asia. In Africa, the most predominant infective agent is Hcc, which can coexist with Hcd. Hcd is primarily found in Central and West Africa and Madagascar, and histoplasmosis caused by this fungus is often referred to as African histoplasmosis, which is a misnomer because African patients can be infected with both variants. Though not endemic in Europe, reports of microfoci of histoplasmosis due to Hcc have been reported in Italy [12].

In the African continent, there are limited incidence cohort data on histoplasmosis despite the burden of HIV disease in sub-Saharan Africa. Surveys of histoplasmin (a mycelial-phase exo-antigen) cutaneous sensitivity have shown that the rate of positive reactors ranges from 0.0% to 28% [13–21], with cross reactivity being demonstrated between Hcc and Hcd in Nigeria [16]. In Nigeria, a higher prevalence of skin test reactivity (approximately 35%) was found in rural populations, especially among farmers, local traders, and cave guides [22].

This review seeks to highlight knowledge gaps regarding the epidemiological, diagnostic (clinical and laboratory), and therapeutic aspects of histoplasmosis in HIV-infected and non–HIV-infected patients in Africa.

## Search strategy and selection criteria

Literature searches for publications on histoplasmosis in Africans preceding 30 March 2017, were performed using PubMed, Web of Science, Google Scholar, Cochrane Library, African Journals Online (AJOL), Africa-Wide: NiPAD, CINAHL (accessed via EBSCO Host) databases, and grey literature to identify all published papers regarding the topic. Articles published in other languages (e.g., French, German, and Portuguese) were considered if they were cited in any of the databases searched. The main search comprised individual searches using detailed medical subject heading (MeSH) terms for histoplasmosis, Africa (also the names of the 54 African countries), and HIV/AIDS combined with terms relevant to histoplasmosis, including broad terms such as ‘diagnosis’ and ‘management’. The Boolean operator ‘AND’ and ‘OR’ were used to combine and narrow the searches. Only reports with patients’ country of origin identified were included. The references in all relevant papers were reviewed for additional publications that may not have been cited elsewhere (‘snow balling’), as well as our own paper files. We did not systematically search all meeting abstracts and other ‘grey literature’, primarily because only a very limited number of scientific conferences related to mycological topics have been held in Africa.

The case definitions employed were based on an international consensus statement by the European Organization for Research and Treatment of Cancer/Invasive Fungal Infections Cooperative Group (EORTC/IFICG) and the Mycoses Study Group (MSG) [23].

## Mycology summary

Historically, *H*. *capsulatum* was considered to be divided into three ‘varieties’ on the basis of morphologic characteristics, as follows: Hcc, prevalent in the Americas; Hcd, mostly reported in Central and Western Africa; and *H*. *capsulatum* var. *farciminosum*, which causes epizootic lymphangitis in horses and is isolated from equines in North Africa and the Middle East [24]. The environmental mould phase (saprophytic form) may form macroconidia and microconidia. Microconidia are smooth-walled with a diameter of 2 to 4 μm and are the infectious elements acquired by inhalation. The yeast phase (parasitic form) develops as oval budding cells with a diameter of 2 to 4 μm, predominantly observed within macrophages and histiocytes, indicative of persistent latency. Hcd yeast forms are larger—8 to 15 μm—with thicker walls, and this differentiation is sufficient for definitive diagnosis [25]. There are other fungi that can be mistaken for *H*. *capsulatum*, for example, *Emmonsia* spp. [26]

## Exposure epidemiology

Prior to the HAART era, subclinical or symptomatic histoplasmosis occurred in 12/100 person-years at risk in a cohort of HIV-infected patients in the US [27]. The introduction of effective ART has not led to a significant reduction in the incidence of histoplasmosis in HIV-infected patients [28]. No such study has been replicated in Africa.

Gugnani and colleagues reported a histoplasmin skin sensitivity prevalence of 3.5% and 3.0% in the community and 8.9% and 6.5% in hospitalised individuals for Hcc and Hcd, respectively, in Nigeria [16]. In Eastern Nigeria, a natural reservoir of Hcd was discovered in the soil of caves mixed with bat guano [22]. There were 20 cases of Hcd infection documented in an outbreak amongst cave explorers in Nigeria [29]. Environmental exposure to nitrogen-rich guano soil has been shown to increase the risk of histoplasmosis in the general populace [27,30]. A landmark study analyzing employment and *Histoplasma* exposure in Uganda in the pre-AIDS era showed that those reactive to histoplasmin were mostly sawmill workers [18]. A past history of cave exploration; presence at and/or participation in excavation sites; woodcutting; and exposure to bird roosts, farms, or poultry have also been documented [31]. In a case-controlled study involving HIV-infected patients with histoplasmosis compared to controls without histoplasmosis, a strong association between histoplasmosis and contact with chicken coops was reported [31]. Skin-test surveys using histoplasmin showed some exposure throughout Central America and parts of South America as well as Puerto Rico, Dominica, and Mexico in addition to the central US with almost no skin-test sensitivity positivity in Europe apart from Italy and France [10].

## Epidemiology of histoplasmosis in Africa

**Fig 1:**
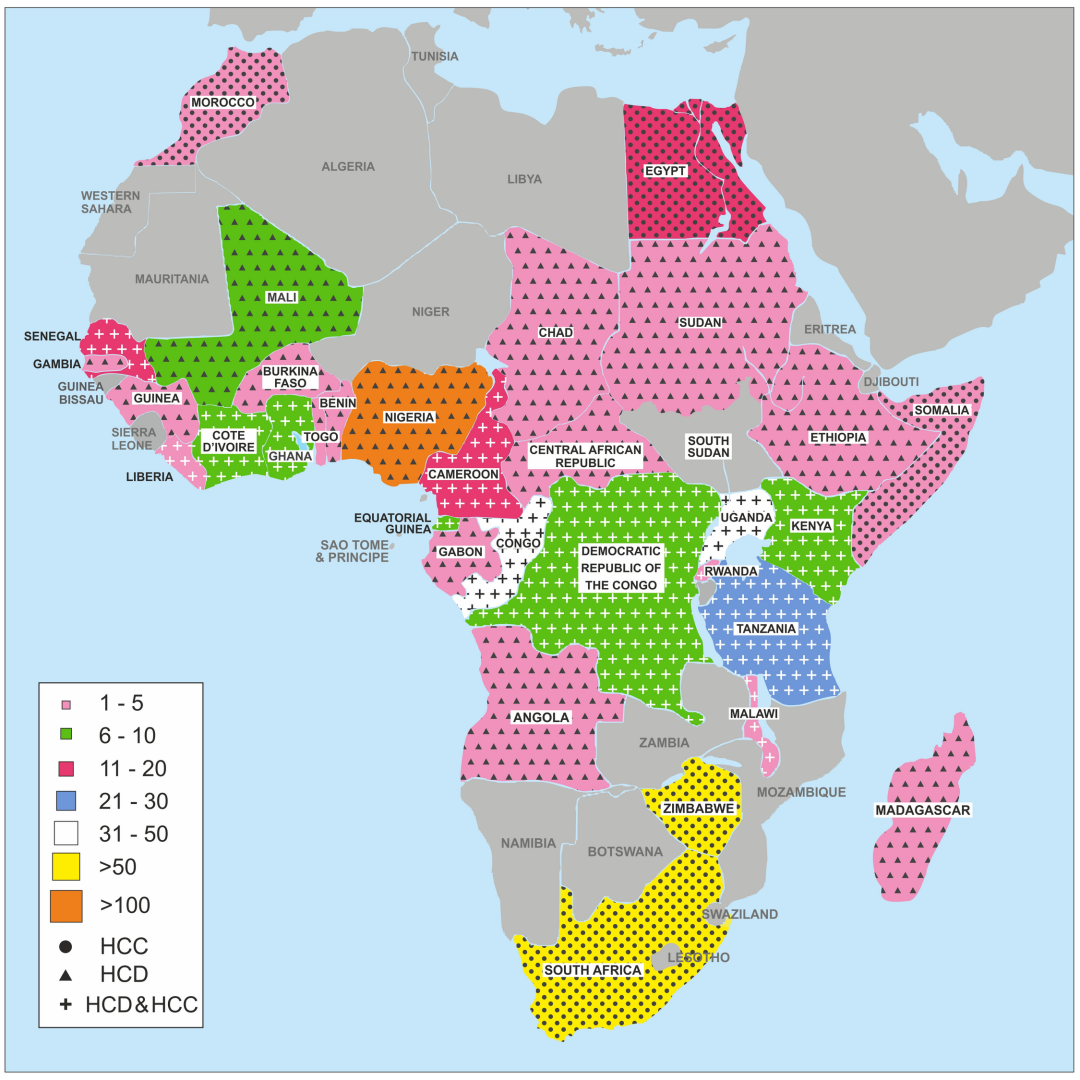
Distribution of reported cases of histoplasmosis across Africa (1952–2017). HCC, *Histoplasma capsulatum* var. *capsulatum*; HCD, *H*. *capsulatum* var. *dubuosii*.

Histoplasmosis—including both Hcd and Hcc—has been reported from 32 countries in Africa (Table 1). While Hcc occurs predominantly in Southern and North Africa, Hcd is found primarily in Central and Western Africa. In addition, there have also been five documented cases described from Madagascar (Fig 1) [32,33]. This map shows the distribution of the reported cases across Africa and not necessarily the true distribution of the burden of the problem. Some African countries lack the skilled personnel and facilities to make the diagnosis, and the number of reported cases is likely to be an underestimation because of the fact that there are few skilled personnel and facilities in many areas.

**Table 1:** Distribution of histoplasmosis in Africa.

Country	Total number of cases	*H*. *capsulatum* var. *dubosii*	*H*. *capsulatum* var. *capsulatum*	HIV positive	HIV negative
W/A					
Nigeria	124	124	-	4	124
Ivory Coast	10	7	3	4	6
Ghana	12	4	8	11	1
Senegal	12	9	3	4	8
Mali	8	8	-	-	8
Liberia	2	1	1	2	-
Gambia	1	1	-	1	=
Togo	2	2	-	2	-
Guinea	2	2	-	-	2
Burkina Faso	3	3	-	-	3
Republic of Benin*	3	1	1	-	3
Mauritania	-	-	-	-	-
Niger	-	-	-	-	-
Guinea Bissau	-	-	-	-	-
Sierra Leone	-	-	-	-	-
Cape Verde	-	-	-	-	-
Summary W/A	179	163	16	28	151
E/A					
Uganda**	36	18	4	3	33
Rwanda	3	2	1	-	3
Kenya	9	2	7	4	4
Ethiopia	1	1	-	?	?
Somali	1	-	1	-	1
Burundi	-	-	-	-	-
South Sudan	-	-	-	-	-
Djibouti	-	-	-	-	-
Eritrea	-	-	-	-	-
Summary E/A	50	23	13	7	41
C/A					
Zaire (DRC)	9	4	5	8	1
Gabon	1	1	-	1	-
Chad	2	2	-	-	2
Central Africa Republic	1	1	-	-	1
Angola	3	3	-	-	3
Congo	36	35	1	23	13
Cameroon	15	6	9	12	3
Equatorial Guinea	6	1	5	3	3
Sao Tome and Principe	-	-	-	-	-
Summary C/A	73	53	20	47	26
S/A					
South Africa***	61	-	61	27	33
Namibia	-	-	-	-	-
Zimbabwe	57	1	56	56	-
Lesotho	-	-	-	-	-
Tanzania****	24	1	1	10	7
Botswana	-	-	-	-	-
Malawi	3	2	1	2	1
Madagascar	5	5	-	-	5
Zambia	-	-	-	-	-
Swaziland	-	-	-	-	-
Mozambique	-	-	-	-	-
Summary S/A	150	9	119	95	46
N/A					
Sudan	1	1	-	-	1
Algeria	-	-	-	-	-
Tunisia	-	-	-	-	-
Morocco	3	-	3	1	2
Libya	-	-	-	-	-
Western Sahara	-	-	-	-	-
Egypt	14	-	14	-	14
Summary N/A	18	1	17	1	17

*One case was based on serological data (histoplasmin).

**13 cases were an outbreak with serological diagnosis and typical clinical pattern; all were pulmonary histoplasmosis.

***HIV testing not done in one case; 15 cases were based on serological testing.

****Majority of diagnoses were based on serological data, and HIV status not stated in some studies.

Abbreviations: C/A, Central Africa; E/A, East Africa; N/A, North Africa; S/A, Southern Africa; W/A, West Africa.

Our exhaustive literature search revealed a total of 470 documented cases of histoplasmosis reported from the African continent (Table 1) dating from 1952 to 2017 (see S1 Table). Hcd accounted for 247 reported cases and Hcc for 185 reported cases, with some only documented serologically. It is noteworthy that a significant number of the histoplasmosis cases in Africa were reported prior to the outbreak of the HIV pandemic. Hcd was the causative organism in osteolytic lesions in HIV-negative children (S1 Table). HIV coinfection was reported predominantly in Hcc and in adults. In contrast to Hcc, HIV and Hcd coinfection was initially thought to be rare in Africans [34,35]. This was most likely due to a problem of underreporting or under-recognition because recent reports have refuted this; our literature search revealed over 25 reported cases (S1 Table).

The distribution of the disease across Africa (Fig 1) is far from uniform; Hcd was predominantly reported in West, Central, and East Africa. The majority of the reports of Hcc were in the southern part of Africa; however, Egypt and Morocco were the only two countries in Northern Africa with reports of human histoplasmosis caused by Hcc. It is possible that some of the Hcc cases in South Africa might have been *Emergomyces africanus*, which is endemic in Southern Africa [36]. Some apparent microfoci of Hcc are reported from the Democratic Republic of Congo (DRC), Ghana, Kenya, Ivory Coast, Benin, and Senegal. Perhaps these cases are due to the mobility and/or emigration of persons in these regions, but probably some microfoci exist. The explanation for the variability and patchy nature of the disease distribution is not clear and could possibly relate to climatic factors, bird migration, and/or contact with bat colonies.

The majority (287 out of 470 [61%]) of the reported cases were in HIV-negative patients. While Hcd accounted for 247 such cases, most were in patients who were immunocompetent (Table 1), and a significant proportion presented as localised cutaneous and/or bone lesions. However, a significant minority presented as disseminated histoplasmosis in AIDS patients. This is consistent with existing data from HIV-infected patients, among which 95% of cases involve disseminated histoplasmosis and 90% of cases involve patients with CD4 counts below 200/mm^3^ [4]. In Southern Africa, there were 119 cases of Hcc diagnosed, with 80% (95) in HIV-infected patients, which is in keeping with a report from Europe that demonstrated the strong relationship between HIV and Hcc coinfection; histoplasmosis was the AIDS-defining disease in nearly 61% of patients in a review of histoplasmosis in Europe [37].

Of the 470 histoplasmosis cases, West Africa had the highest number of cases (179 [38%]) followed by Southern Africa (150 [32%]), while Northern Africa had the fewest documented cases (18) (Fig 1). In contrast, there are several cases of equine histoplasmosis from Northern Africa [38]. Nigeria had the highest number of reported cases (124 cases) (Table 1). Most cases presented as cutaneous and bone lesions, with only four cases of disseminated Hcd histoplasmosis, which were all HIV-positive patients. These four patients were Nigerian émigrés in the Western world and Saudi Arabia. Nigeria has the second highest number of people living with HIV infection in Africa, an estimated three million [39]. Interestingly, in several of the case reports from Nigeria, patients were first misdiagnosed as malignancies until the histology report showed otherwise. In contrast, South Africa reported only Hcc cases, with almost an even distribution between HIV-positive and -negative patients. Zimbabwe, which shares a border with South Africa, had 57 histoplasmosis cases reported in one series, all Hcc apart from one Hcd case.

## Clinical presentation

While Hcc typically presents as an acute respiratory or febrile picture, Hcd rarely manifests as pulmonary disease but more commonly as a subacute infection of skin, lymph nodes, subcutaneous (abscesses), and bone lesions. Disease manifestations vary depending on immune status and the number of fungal particles inhaled. Most cases of acute histoplasmosis in immunocompetent individuals tend to resolve spontaneously [11]. In immunocompromised patients, histoplasmosis accounts for significant morbidity and mortality [6].

## Hcd infection

A large case series (56 patients) by Cockshott and Lucas gave a detailed clinical presentation of Hcd infection [40]. Cutaneous, subcutaneous, and bone lesions were the most common clinical presentations of this infection and can be localised or disseminated [40]. Cutaneous histoplasmosis is considered to be from haematogenous spread, although occasionally primary inoculation of the skin has been documented [35]. In a series of ‘African histoplasmosis’ patients from Mali, 62% presented with skin diseases, 46% with lymphadenopathy, 21% with bone lesions, 26% with gut disease, and 4% with infection of the lungs [41]. The report of 72 patients with AIDS from Europe revealed skin manifestations in 47.2% of all cases; among the 27 cases acquired from Africa—of which seven were cases of Hcd—skin manifestations were seen in 44% [37]. Localised cutaneous Hcd is seen most predominantly in the immunocompetent individual, while disseminated Hcd is seen in the immunosuppressed. However, disseminated cutaneous disease has been documented in immunocompetent individuals infected with African histoplasmosis in Africans and non-Africans [42]. The spectrum of cutaneous eruption includes polymorphic plaques, papules, pustules, nodules, ulcers, molluscum-like lesions, acneiform eruptions, exfoliative erythroderma, abscesses, and cellulitis irrespective of the immune status [43].

Gastrointestinal histoplasmosis from Hcd has been reported in disseminated disease and the progressive disseminated form. It was found to be more common in the setting of HIV in the review by Loulergue and colleagues [44]. Clinical features include abdominal pain, hematemesis, diarrhoea, dysphagia from peritonitis, gastrointestinal bleeding, and intestinal pathologies such as ulceration and perforation [37,44–46]. Table 2 summarises the presentation of histoplasmosis in Africans.

**Table 2:** Clinical presentation of histoplasmosis in Africans.

Clinical Classification	Presentations	Details/Complications	Outcome/Comments	References
• Asymptomatic disease • Frequently seen	• Positive skin sensitivity and serology • Skin and lymphadenopathy (localised)	• No symptoms • Seen in areas of low exposure and in the immune-competent	• Nonprogressive skin infection (mainly in Hcd)	[34,35]
• Localised disease • Common (mostly in competent; however, seen sometimes in immune-suppressed)	• Skin and subcutaneous	• Cutaneous lesions include ulcers, molluscum-like nodules and papules, nonhealing ulcers	• Good outcome • Clinical remission and clearing • Most common feature in Hcd • No systemic involvement and lesions are scanty	[37,42,43,86,87]
	• Lymphadenopathy		Can occur in Hcd and Hcc	[85]
	• Lungs—diffuse or nodular infiltrates (occasionally)	Often asymptomatic	• Common in Hcc but rare in Hcd, though has been documented	[37,88]
• Disseminated disease • Not rare • Common in immune-suppressed (HIV, myelosuppressive disorders, cytotoxics, etc.) • Sometimes seen in immune-competent	• Fever and constitutional symptom	• A cause of pyrexia of undetermined origin	• Good outcome with early diagnosis and institution of HAART	[51,87,89–92]
	Pulmonary disease • APH	• May be asymptomatic; flu-like, fever, dry cough, chest pain, headaches, EN, or EM; patchy pneumonia or nodular shadows on CXR	• Good outcome if diagnosed early • May be self-limiting but severe, with large inoculum hence prompt diagnosis (occurs in Hcc, rare in Hcd)	[51,64,93]
	• SAPH	• Asymptomatic complications are pericarditis with pleural effusion and pulmonary fibrosis	• Presentation is usually late in Africans; initial misdiagnosis of TB or sarcoidosis • Outcome often poor with death and disability (mostly in Hcc) • Some documented in Hcd • EN and EM signify dissemination • Good outcome with early diagnosis and institution of HAART (for HIV-positive patients)	[51,87,89–92]
• Chronic histoplasmosis	• CPH	• Cough, haemoptysis, dyspnoea, and chest pain • Cavitation, reticulonodular shadows, apical lesions, hilar adenopathy, progressive fibrosis, and cor pulmonale	• Found mainly in Hcc; clinical features similar to TB	[37, 51]
	• Lymph nodes	• Hilar, cervical, axillary • Granulomatous disease and mediastinitis local compression	• Common in both Hcc and Hcd	[44–46]
	Extra pulmonary disease • CNS	• Lowered consciousness, acute or chronic meningitis, headaches, CN deficit, stroke-like, seizures, confusion	• Mostly Hcc, almost nonexistent with Hcd	[37,94]
	Gastrointestinal	• Peritonitis, perforation, hepatomegaly, and splenomegaly	• Most common with Hcc, rarely with Hcd	[37,44–46]
	• Rheumatologic	• Symmetrical arthralgia of knees, ankle, and elbow + EN	• Mostly Hcc, rarely Hcd	[11,95]
	• Hematologic	• Anaemia, leukopaenia, pancytopenia	• Both Hcc and Hcd in immune-suppressed, Hcc most commonly	[11]
	• Ocular	• Atrophic choroidal spots, peripapillary pigment, and maculopathy	• Found in previous Hcc and those living in endemic areas • Not described in Hcd	[96,97]
	• Pericarditis	• Occurs with pulmonary infection	• Mainly Hcc, rarely Hcd	[37]
	• Cutaneous	• Features described in localised + tumours, abscesses	• More extensive than the localised form and may have other systemic disease	[37,42,43,86,87]
	• Adrenal affectation	• Nonspecific symptoms such as recurrent fever, weight loss, anorexia + skin pigmentation • Asymptomatic as adrenal mass	• Outcome good with early management • May or may not cause adrenal insufficiency (mainly in Hcc)	[98–100]
• PDH • Risk factors include: AIDS, use of corticosteroids, haematological malignancies, solid organ transplant, use of TNFα inhibitors (infliximab and etenercept)	• Constitutional symptoms (found in acute and chronic PDH) • Gastrointestinal tract involvement (subacute PDH) • Cardiac • CNS • Mucosal affectation	• Weight loss, fever, malaise, dyspnoea • Diarrhoea and abdominal pain (as in disseminated disease) • Valvular disease, cardiac insufficiency, vegetation: dyspnoea, peripheral oedema, angina, and fever • Headaches, visual, gait disturbance, confusion, seizures, altered consciousness, neck stiffness and pain	• Very poor outcome (rarely Hcd, mostly Hcc) • Diarrhoea and abdominal pain implies chronic progressive disease and diagnostic clue	[44–46]

Abbreviations: APH, acute pulmonary histoplasmosis; CN, cranial nerve; CNS, central nervous system; CPH, chronic pulmonary histoplasmosis; CXR, chest X-ray; EM, erythema multiforme; EN, erythema nodusum; HAART, highly active antiretroviral therapy; Hcc, *Histoplasma capsulatum* var. *capsulatum*; Hcd, *H*. *capsulatum* var. *dubuosii*; PDH, progressive disseminated histoplasmosis; SAPH, subacute pulmonary histoplasmosis; TB, tuberculosis; TNFα, tumour necrosis factor alpha.

## Histoplasmosis and TB

TB is the closest mimic of histoplasmosis, and limited access to diagnostic facilities may be responsible for some misdiagnoses. Similar to pulmonary TB, chronic pulmonary histoplasmosis (usually Hcc) starts with malaise, fever, fatigue, cough, and sputum. However, sputum production, weight loss, and night sweat are less prominent than TB [47]. Eventually, chronic pulmonary histoplasmosis often results in pulmonary insufficiency and cor pulmonale. It rarely causes death if untreated, unlike TB at the advanced stage [47,48].

In the context of HIV infection, comparative studies of TB and histoplasmosis found that whilst many similar features are noted in the two infections, with most reports detailing information on disseminated Hcc infection, there are also some distinguishing features (Table 3) [7]. In the HIV/AIDS context, disseminated histoplasmosis is usual, often with some pulmonary involvement, but other clinical features are more prominent, notably diarrhoea [49] or other gastrointestinal symptoms, skin lesions, and pancytopenia, which vary in different regions. Coinfection of TB and histoplasmosis has been documented in 8% to 15% of cases [50]. Other features include reticulonodular lesions, multiple pulmonary nodules, hilar and mediastinal adenopathy, and progressive fibrosis [37,51].

**Table 3:** Summary of major features associated with tuberculosis and disseminated histoplasmosis in context of HIV infection.

Parameters	Tuberculosis	Histoplasmosis
Immune suppression	↓↓	↑↑
Pancytopaenia	↓↓	↑↑
Diarrhoea	↓↓	↑↑
Renal function	––	––
High liver function tests (AST, ALT, γGT, ALP, LDH)	↓↓	↑↑
Hepatosplenomegaly (clinical and ultrasound)	↓↓	↑↑
Inflammatory markers (CRP >70 mg/L)	––	––
Ferritin and triglyceride	↓↓	↑↑
Systemic involvement (GIT, bone marrow, liver and peripheral blood)	↓↓	↑↑
Disseminated disease	↓↓	↑↑
Respiratory system (clinical and laboratory)	↑↑	↓↓
Central nervous system involvement (clinical and investigation)	––	––
Skin	↓↓	↑↑

––, Similar features; ↓↓, Lower frequency; ↑↑, Higher frequency.

## Diagnosis

Culture is potentially hazardous for laboratory personnel and requires a level 3 biosafety facility (which is not available in most African countries). It remains the reference method for disseminated histoplasmosis, particularly in HIV-infected patients, although growth requires a one- to six-week incubation, resulting in delay of treatment initiation [52]. Though specificity of the culture method is 100%, sensitivity depends on the fungal load. Bone marrow aspirates yield the highest proportion of positive cultures (70%–90%) [53]. Culture is not very useful in diagnosing primary infections in immunocompetent patients with low fungal load [54]. In disseminated cases, blood cultures using the centrifugation–lysis system or automated blood culture systems have increased sensitivity [55,56].

Bone marrow biopsy for histopathology is a rapid method of establishing a definitive diagnosis of disseminated histoplasmosis [57]. However, this method lacks sensitivity for subacute and chronic forms of the pulmonary histoplasmosis [57]. Another challenge with histology is that the morphology of the *H*. *capsulatum* yeasts is very similar to other pathogens, and these characteristics can lead to a mistake in identification [58]. Misidentification occurs principally with *Candida glabrata*, *Talaromyces marneffei*, *Pneumocystis jirovecii*, *Toxoplasma gondii*, *Leishmania donovani*, and *Cryptococcus neoformans*.

Serology for anti-*Histoplasma* antibodies is particularly useful in cases of low fungal load, as in asymptomatic or chronic pulmonary histoplasmosis [52]. Antibody detection by immunodiffusion or complement fixation is less sensitive in immunocompromised HIV-infected patients than in immunocompetent patients [59,60]. The rise of antibody titres is usually observed two to six weeks after primary exposure [10]. Antibody testing of cerebrospinal fluid (CSF) is critical for suspected cases of neuromeningeal histoplasmosis [61]. Cross-reactions with other fungal pathogens, lymphoma, sarcoidosis, and TB have been reported [62]. A recent study from Brazil using a western blot test strip found a sensitivity of 94.9%, specificity of 94.1%, positive predictive value (PPV) 94.1%, negative predictive value (NPV) 94.9%, and almost perfect precision [63]. Another recent study demonstrated that the Miravista *Histoplasma* antibody enzyme immunoassay (EIA) offers increased sensitivity over other current antibody tests and detects both immunoglobulin G (IgG) and IgM antibodies and complements antigen detection [64]. Therefore, combining antigen and EIA antibody testing provides an optimal method for diagnosis of acute pulmonary histoplasmosis [64].

Diagnosing disseminated histoplasmosis has been significantly facilitated by the development of *Histoplasma* antigen testing. The detection of Hcc circulating antigen has been performed using several EIA methods. Antigen testing of blood or urine in disseminated histoplasmosis is most sensitive in immunocompromised patients and those with more severe illness, with higher titres [65,66]. The antigen level correlates with the severity of the disease [67]. *Histoplasma* antigen cross-reacts in sporotrichosis [68,69], aspergillosis (10%), coccidioidomycosis (60%), paracoccidioidomycosis (80%), and blastomycosis (90%) [67,68]. In spite of this, *Histoplasma* antigen testing still remains the mainstay of diagnosing histoplasmosis in immunocompromised patients. There is no cross-reactivity between *C*. *neoformans* and *Histoplasma* antigen [70,71].

Molecular methods have been reported for the diagnosis of histoplasmosis with inconsistent accuracy [59,72–74]. No single approach based on nucleic acid amplification assays has been established as the dominant method; *Histoplasma* can be detected in tissue biopsies and whole blood, but these methods are not sensitive enough to identify *Histoplasma* in urine or serum [75,76].

In most of the reported cases from Africa, the diagnosis was made by culture and histology; only in five countries (Tanzania, Benin, South Africa, Egypt, and Uganda) was serology reported as being used to make a diagnosis, and in three of the cases, the samples were processed in Western countries.

## Treatment

The Infectious Diseases Society of America recommends prophylaxis with itraconazole for as long as the CD4 count remains below 150/mm^3^ in highly endemic areas with an incidence of histoplasmosis of >10 cases per 100 person-years [77]. If efavirenz and itraconazole are given together, itraconazole levels fall by 40%, so higher doses are required.

While waiting for laboratory confirmation of histoplasmosis in a patient with a strong suspicion of histoplasmosis with or without severe symptoms, physicians have the following two choices for treatment induction: intravenous (IV) amphotericin B or oral itraconazole [53]. Although amphotericin B is usually fungicidal and has shown its efficacy in terms of survival, it is also nephrotoxic [78]. Liposomal amphotericin is superior to conventional amphotericin B for disseminated histoplasmosis in AIDS [78]. Itraconazole is also fungicidal for most isolates of *H*. *capsulatum*, but oral capsules are not always well absorbed in advanced AIDS, and it is associated with many drug–drug interactions, including rifampicin. For these reasons, amphotericin B is preferred for initial therapy of disseminated histoplasmosis in AIDS, but itraconazole is a good choice for subacute disseminated infection [53].

Worryingly, amphotericin B is not licensed and is unavailable in a number of African countries; even where it is available, the cost may be prohibitive [79]. Liposomal amphotericin B is excessively costly and not available in most of Africa. While available in most African countries, itraconazole is prohibitively costly in most [79]. Generic formulations are available, but varying quality is a challenge [80].

Clinical outcomes were variable in the African studies reviewed and depended on a number of factors, such as the type of disease, early/prompt diagnosis, and accessibility to the effective drugs. Most cases of cutaneous lesions and disseminated diseases resolve with amphotericin B and itraconazole, while response to ketoconazole was variable and often poor [81–85]. Loulerge and colleagues reported good responses (>50%) and cure in disseminated HIV-positive cases mainly with amphotericin B and high-dose itraconazole [44]. In cases with HIV, outcomes were complicated by other comorbidities associated with immune suppression [46]. In AIDS patients in the US, histoplasmosis-related mortality was around 10% during the HAART era [52].

In conclusion, histoplasmosis is a neglected disease in Africa, a continent that has a significant number of people living with HIV/AIDS. Under-recognition and under-diagnosis are major challenges attributable to the lack of skilled personnel and facilities to make this diagnosis. It is imperative that concerted efforts be made in tackling this. It is also important for physicians outside of endemic regions to recognise this disease and how to manage it. This is particularly important in view of migratory patterns of Africans.

**Key learning points**

- Histoplasmosis is a neglected disease in Africa. This is because histoplasmosis in Africa has markedly increased but is under-recognised.
- Histoplasmosis may be misdiagnosed as TB.
- Africa has a significant number of people living with HIV/AIDS, which is the greatest attributable risk factor for histoplasmosis.
- The ongoing health agenda for Africa must acknowledge the lack of skilled personnel and facilities to make the diagnosis of histoplasmosis in Africa.
- There is limited accessibility and availability of antifungal agents on the African continent.

**Top five papers**

1. Wheat LJ. Histoplasmosis: A review for clinicians from non-endemic areas. Mycoses. 2006;49(4):274–82.
2. Hage CA, Ribes JA, Wengenack NL, Baddour LM, Assi M, McKinsey DS, et al. A Multicenter Evaluation of Tests for Diagnosis of Histoplasmosis. Clin Infect Dis 2011;53(5):448–54
3. Antinori S, Magni C, Nebuloni M, Parravicini C, Corbellino M, Sollima S, et al. Histoplasmosis Among Human Immunodeficiency Virus-Infected People in Europe. Medicine (Baltimore) 2006;85(1):22–36.
4. Kneale M, Bartholomew JS, Davies E, Denning DW. Global access to antifungal therapy and its variable cost. J Antimicrob Chemother 2016;71(12):3599–606.
5. Valero C, Gago S, Monteiro MC, Buitrago MJ. African histoplasmosis: new clinical and microbiological insights. 2017; Med Mycol. 2018;56(1):51-59. doi: 10.1093/mmy/myx020

## Supporting information

S1 Table Reported cases of histoplasmosis in Africa (1952–2017). (DOCX)
