# Supplementary material for: Histoplasmosis in Africa: An emerging or a neglected disease?
Source: PLoS Negl Trop Dis. 2018 Jan 18;12(1):e0006046. doi: 10.1371/journal.pntd.0006046 (PMC5773084; doi:10.1371/journal.pntd.0006046)
Supplement: S1 Table — (DOCX) [file pntd.0006046.s001.docx]

| **Country** | **Type of disease and causative agent** | **HIV status** | **Reference** | |
| --- | --- | --- | --- | --- |
| Garbon | Cutaneous histoplasmosis; Hcd | Positive | [1] | |
| Egypt | Disseminated histoplasmosis;Hcc | Negative | [2] | |
| Egypt | Pulmonary histoplasmosis; Hcc (13cases) | Negative | [3] | |
| Zimbabwe | Cutaneous histoplasmosis (12 cases);Hcd | Positive | [4] | |
| Chad | cutaneous and subcutaneous  abscesses in child; Hcd | Negative | [5] | |
| Chad | Pulmonary histoplasmosis; Hcd | Negative | [6] | |
| Ivory coast | Cutaneous histoplasmosis in child; Hcd | Negative | [7] | |
| Ivory coast | Ganglionic histoplasmosis; Hcd | Negative | [8] | |
| Ivory coast | Cutaneous histoplasmosis; Hcd | Positive | [9] | |
| Ivory coast | Cutaneous and bone lesions; Hcd | Negative | [10] | |
| Ivory coast | Cervical adenopathy,IRIS ; Hcc | Positive | [11] | |
| Ivory coast | Disseminated histoplasmosis in a child ;Hcc | Negative | [12] | |
| Ivory coast | Disseminated histoplasmosis;Hcd | Negative | [13] | |
| Ivory coast | Disseminated histoplasmosis (2cases); Hcc | Positive | [14] | |
| Ivory coast | Disseminated histoplasmosis; Hcc | Positive | [15] | |
| Ivory coast | Cutaneous histoplasmosis; Hcd | Negative | [16] | |
| Zaire DRC | Disseminated histoplasmosis; Hcc | Positive | [17] | |
| Zaire DRC | Disseminated histoplasmosis in child; Hcc | Not stated | [18] | |
| Zaire DRC | Disseminated histoplasmosis (4 cases); Hcd | (1)Positive | [19] | |
| Zaire | Disseminated histoplasmosis (3 Cases) ;Hcc | Positive | [20] | |
| Ghana | Disseminated histoplasmosis; Hcc | Positive | [21] | |
| Ghana | Disseminated histoplasmosis ;Hcc | Positive | [22] | |
| Ghana | Histoplasmosis colitis;Hcc | Positive | [23] | |
| Ghana | Pulmonary histoplasmosis; Hcc | Negative | [24] | |
| Ghana | Disseminated histoplasmosis ;Hcc | Positive | [25] | |
| Ghana | Disseminated histoplasmosis ;Hcc | Positive | [15] | |
| Ghana | Disseminated histoplasmosis ;Hcc | Positive | [26] | |
| Ghana | Disseminated histoplasmosis ;Hcc | Positive | [27] | |
| Ghana | Disseminated histoplasmosis;Hcd | Positive | [28] | |
| Ghana | Disseminated histoplasmosis;Hcd | Positive | [29] | |
| Senegal | Disseminated histoplasmosis; Hcc | Negative | [30] | |
| Senegal | Disseminated histoplasmosis;Hcd | Negative | [31] | |
| Senegal | Disseminated histoplasmosis;Hcd | Positive | [28] | |
| Senegal | Disseminated histoplasmosis; Hcc | Positive | [15] | |
| Senegal | Disseminated histoplasmosis;Hcd | Positive | [32] | |
| Senegal | Disseminated histoplasmosis; Hcc | Positive | [33] | |
| Senegal | Cutaneous histoplasmosis; Hcd | Negative | [34] | |
| Senegal | Cutaneous histoplasmosis; Hcd | Negative | [35] | |
| Senegal | Disseminated histoplasmosis;Hcd | Negative | [36] | |
| Mali | Disseminated histoplasmosis;Hcd | Negative | [37] | |
| Mali | Subcutaneous and bone histoplasmosis; Hcd | Negative | [38] | |
| Mali | Cutaneous histoplasmosis; Hcd | Negative | [39] | |
| Mali | Bone lesion in a child; Hcd | Negative | [40] | |
| Mali | Disseminated histoplasmosis (4 cases); Hcd | Negative | [41] | |
| Congo | Cutaneous histoplasmosis; Hcd | Negative | [42] | |
| Congo | Histoplasmosis bone lesion; Hcd | Negative | [43] | |
| Congo | Disseminated histoplasmosis;Hcd | Positive | [44] | |
| Congo | Cutaneous histoplasmosis; Hcd | Negative | [45] | |
| Congo | Histoplasmosis bone lesion; Hcd | Negative | [43] | |
| Congo | Disseminated histoplasmosis;Hcd | Positive | [46] | |
| Congo | Disseminated histoplasmosis;Hcc | Positive | [47] | |
| Congo | Disseminated histoplasmosis;Hcd | Positive | [48] | |
| Congo | Histoplasmosis bone lesion ;Hcd | Negative | [49] | |
| Congo | Cut, bone and ganglionic lesions (13cases); Hcd | Negative | [50] | |
| Congo | Histoplasmosis lymphadenopathy; Hcd | Negative | [51] | |
| Congo | Cutaneous histoplasmosis (2cases);Hcd | Negative | [52] |  |
| Congo | Disseminated histoplasmosis (3 cases);Hcd | Positive | [53] | |
| Congo | Disseminated histoplasmosis; Hcd | Positive | [54] | |
| Congo | Disseminated histoplasmosis (6 cases, 1 child); Hcd | 4 Positive  2 Negative | [55] | |
| Congo | Disseminated histoplasmosis; Hcd | Positive | [56] | |
| Congo | ? Disseminated histoplasmosis (11cases); Hcd | Positive | [57] | |
| Congo | Disseminated histoplasmosis (3 peadiatric cases);Hcd | Negative | [58] | |
| Nigeria | Disseminated histoplasmosis (2cases); Hcd | Positive | [28] | |
| Nigeria | Disseminated histoplasmosis;Hcd | Positive | [59] | |
| Nigeria | Histoplasmosis bone lesion; Hcd | Negative | [60] | |
| Nigeria | Disseminated histoplasmosis in a child; Hcd? | Negative | [61] | |
| Nigeria | Cutaneous histoplasmosis; Hcd | Negative | [62] | |
| Nigeria | Histoplasmosis lymphadenopathy; Hcd | Negative | [63] | |
| Nigeria | Subcutaneous histoplasmosis; Hcd | Negative | [64] | |
| Nigeria | Cutaneous histoplasmosis(52 cases); Hcd | Negative | [65] | |
| Nigeria | Histoplasmosis, jenunal lesion; Hcd | Negative | [66] | |
| Nigeria | Histoplasmosis, bone lesions (6 Cases) in children; Hcd | Negative | [67] | |
| Nigeria | Histoplasmosis, vocal cord lesion | Negative | [68] | |
| Nigeria | Histoplasmosis, oral and bone lesions;Hcd | Negative | [69] | |
| Nigeria | Cutaneous histoplasmosis; Hcd | Negative | [70] | |
| Nigeria | Histoplasmosis, oral lesions | Negative | [71] | |
| Nigeria | Histoplasmosis, bone lesions; Hcd | Negative | [72] | |
| Nigeria | Histoplasmosis bone lesions; Hcd | Negative | [73] | |
| Nigeria | Histoplasmosis colonic mass; Hcd | Negative | [74] | |
| Nigeria | Cutaneous histoplasmosis (12 cases); Hcd | Negative | [75] | |
| Nigeria | Histoplasmosis, bone lesion in child;Hcd | Negative | [76] | |
| Nigeria | Cutaneous and subcutaneous histoplasmosis (2cases); Hcd | Negative | [77] | |
| Nigeria | Cutaneous and osteolytic hisplasmosis (2 adults and 2children); Hcd | Negative | [78] | |
| Nigeria | Subcutaneous histoplasmosis (3cases); Hcd | Negative | [79] | |
| Nigeria | Histoplasmoisi, oral lesion in a child; Hcd | Negative | [80] | |
| Nigeria | Cutaneous and osteolytic histoplasmosis in an child: Hcd | Negative | [81] | |
| Nigeria | Cutaneous and osteolytic hisplasmosis | Negative | [82] | |
| Nigeria | Disseminated histoplasmosis in 2 children | Negative | [83] | |
| Nigeria | Histoplasmosis bone lesion; Hcd | Negative | [84] | |
| Nigeria | Cutaneous histoplasmosis (10 cases) | Negative | [85] | |
| Nigeria | Histoplasmosis, bone lesion; Hcd | Negative | [86] | |
| Nigeria | Pulmonary histoplasmosis (2cases); Hcd | Negative | [87] | |
| Nigeria | Histoplasmosis orbital cyst; Hcd | Negative | [88] | |
| Nigeria | Histoplasmosis orbital lesion in a child; Hcd | Negative | [89] | |
| Nigeria | Histoplasmosis, skull bone lesion: Hcd | Negative | [90] | |
| Gambia | Disseminated histoplasmosis: ?Hcd | Positive | [91] | |
| Liberia | Cutaneous histoplasmosis: Hcd | Positive | [92] | |
| Liberia | Disseminated histoplasmosis; Hcc | Positive | [15] | |
| Togo | Histoplasmosis, bone lesions in a child; Hcd | Negative | [93] | |
| Togo | Histoplasmosis lymphadenopathy; Hcd | Positive | [94] | |
| CAR | Histoplasmosis; bone and joint lesions: Hcd | Positive | [95] | |
| Madazascar | Pulmonary histoplasmosis: Hcd | Negative | [96] | |
| Madagascar | Histoplasmosis (3cases); Hcd |  | [97] | |
| Equatorial Guinea | Oral histoplasmosis; Hcc | Negative | [98] | |
| Equatorial Guinea | Cutaneous histoplasmosis; Hcc | Positive | [99] | |
| Equatorial Guinea | Pulmonary histoplasmosis; Hcc | Negative | [100] | |
| Equatorial Guinea | Disseminated Histoplasmosis; Hcd | Positive | [28] | |
| Guinea | Cutaneous histoplasmosis ;Hcd | Negative | [101] | |
| Guinea | Histoplasmosis lymphadenopathy: Hcd | Negative | [102] | |
| Sudan | Cutaneous histoplasmosis ;Hcd | Negative | [103] | |
| Ethiopia | Histoplasmosis, cutaneous and bone lesions; Hcd | Negative | [104] | |
| Kenya | Disseminated histoplasmosis (4cases) 2 children; Hcc | 3 Positive | [105] | |
| Kenya | Intestinal histoplasmosis; Hcd | Negative | [106] | |
| Kenya | Disseminated histoplasmosis in a child;Hcc | Negative | [107] | |
| Kenya | Disseminated histoplasmosis; Hcc | Positive | [108] | |
| Kenya | Disseminated histoplasmosis in a child; ?Hcd | Negative | [109] | |
| Kenya | Pulmonary histoplasmosis; Hcc | Negative | [110] | |
| South African | Disseminated histoplasmosis; Hcc | Negative | [111] | |
| South Africa | Disseminated cutaneous histoplasmosis (14cases); Hcc | Positive | [112] | |
| South Africa | Pulmonary histoplasmosis (10 cases); Hcc | Negative | [113] | |
| South Africa | Histoplasmosis, oral lesions(2cases);Hcc | Positive | [114] | |
| South Africa | Disseminated histoplasmosis; Hcc | Negative | [115] | |
| South Africa | Disseminated histoplasmosis in a child; Hcc | Positive | [116] | |
| South Africa | Disseminated histoplasmosis (3 cases); Hcc | Positive | [117] | |
| South Africa | Disseminated histoplasmosis | Negative | [118] | |
| South Africa | Pulmonary histoplasmosis (20 cases); histoplasmin antigen testing | Negative | [119] | |
| South Africa | Disseminated histoplasmosis; Hcc | Positive | [120] | |
| South Africa | Disseminated histoplasmosis; Hcc | Negative | [121] | |
| South Africa | Disseminated histoplasmosis (2cases); Hcc | Negative | [122]. | |
| South Africa | Disseminated histoplasmosis; Hcc | Positive | [123] | |
| South Africa | Histoplasmosis, oral lesions; Hcc | Negative | [124] | |
| South Africa | Disseminated histoplasmosis in a child; Hcc | Positive | [125] | |
| South Africa | Histoplasmosis, intestinal lesions: Hcc | Negative | [126] | |
| South Africa | Histoplasmosis, oral lesions:Hcc | Positive | [127] | |
| Morocco | Cutaneous histoplasmosis; Hcc | Negative | [128] | |
| Morocco | Histoplasmosis sinusitis; Hcc | Positive | [129] | |
| Morocco | Histoplasmosis bone lesion; Hcc | Negative | [130] | |
| Malawi | Disseminated histoplasmosis; Hcd | Negative | [131] | |
| Malawi | Histoplasmosis, bone lesion ;Hcd | Positive | [132] | |
| Malawi | Disseminated histoplasmosis;Hcc | Positive | [133] | |
| Rwanda | Pulmonary histoplasmosis;Hcc | Negative | [134] | |
| Rwanda | Cutaneous histoplasmosis; Hcd | Negative | [135] | |
| Rwanda | Cutaneous histoplasmosis; Hcd | Negative | [136] | |
| Angola | Cutaneous histoplasmosis; Hcd | Negative | [137] | |
| Angola | Cutaneous and lymph node histoplasmosis; Hcd | Negative | [138] | |
| Angola | Cutaneous histoplasmosis and lymphadenopathy; Hcd | Negative | [139] | |
| Somali | Oral histoplasmosis; Hcc | Negative | [140] | |
| Tanzania | Pulmonary histoplasmosis (3 cases) | Not stated | [141] | |
| Tanzania(Moshi) | Disseminated histoplasmosis (9cases); serology | 6 Positive  3 Negative | [142] | |
| Tanzania | Pulmonary histoplasmosis in child; Hcc | Negative | [143] | |
| Tanzania (Moshi) | 4 adults cases of Histoplasmosis (4cases) serology | 3 Positive  1 Negative | [144] | |
| Tanzania (Moshi) | Histoplasmosis 2 children; serology | 1 Positive  1 Negative | [145] | |
| Tanzania | Oral histoplasmosis lesion; Hcc |  | [146] | |
| Tanzania (Tanganyika) | Disseminated histoplasmosis;Hcd | Negative | [147] | |
| Benin | Disseminated histoplasmosis (3cases); 1Hcc, 1 Hcd, 3^rd^ serology | Negative | [148] | |
| Uganda | Spinal histoplasmosis; Hcd | Negative | [149] | |
| Uganda | Histoplasmosis bone lesions in a child; Hcd | Negative | [150] | |
| Uganda | Cutaneous,, lymph nodes and/or bone abnormalities; Hcd | Negative | [151] | |
| Uganda | Pulmonary histoplasmosis |  | [152] | |
| Uganda | Oral histoplasmosis lesions; Hcc | Positive | [153] | |
| Uganda | Pulmonary histoplasmosis (13 cases) | Negative | [154] | |
| Uganda | Disseminated histoplasmosis;Hcd | Negative | [155] | |
| Uganda | Cutaneous histoplasmosis;Hcc | Positive | [156] | |
| Uganda | Mesenteric cyst with histoplasmosis in a child; Hcc | Negative | [157] | |
| Uganda | Cutaneous histoplasmosis,IRIS; Hcc | Positive | [158] | |
| Burkina Faso | Disseminated histoplasmosis;Hcd | Negative | [159] | |
| Burkina Faso | Disseminated histoplasmosis in a child, cutaneous and subcutaneous in an adult; Hcd | Negative | [160] | |
| Cameroon | Disseminated histoplasmosis in a child; Hcd | Negative | [161] | |
| Cameroon | Disseminated histoplasmosis (7cases); Hcc | Positive | [162] | |
| Cameroon | Pulmonary histoplasmosis; Hcd | Negative | [163] | |
| Cameroon | Buccal lesion; Hcd | Negative | [164] | |
| Cameroon | Disseminated histoplasmosis; Hcc | Positive | [165] | |
| Cameroon | Disseminated histoplasmosis; Hcc | Positive | [166] | |
| Cameroon | 2 cases (1 lymphadenopathy, 1 Buccal lesions) Hcd and Hcc respectively | Negative | [167] | |
| Cameroon | Disseminated histoplasmosis; Hcd | Positive | [168] | |

References

1. Mounguengui D, Ondounda M, Mandji Lawson JM, Coniquet S, Moussounda M, Magne C, et al. Histoplasmose: Premi??re observation au Gabon. Med Sante Trop. 2012;22: 82–83. doi:10.1684/mst.2012.0015

2. Elbadawi A, Ahmed HMA, Adly H, Elkhouly MA, Abohamed S, Falsey AR. Acute Disseminated Histoplasmosis with Atypical Lymphocytosis in an Immunocompetent Host. IDCases. 2017;7: 23–24. doi:10.1016/j.idcr.2016.11.006

3. Moch RW, Walker PF, Kerkor M, Botros BA. Human histoplasmosis in Egypt-a preliminary serologic survey. J Trop Med Hyg. 1976;79: 244–6. Available: http://www.ncbi.nlm.nih.gov/pubmed/1011320

4. Gumbo T, Just-Nübling G, Robertson V, Latif AS, Borok MZ, Hohle R. Clinicopathological features of cutaneous histoplasmosis in human immunodeficiency virus-infected patients in Zimbabwe. Trans R Soc Trop Med Hyg. 2001;95: 635–636. doi:10.1016/S0035-9203(01)90103-8

5. Garcia-Guiñon A, Torres-Rodríguez JM, Ndidongarte DT, Cortadellas F, Labrín L. Disseminated histoplasmosis by Histoplasma capsulatum var. duboisii in a paediatric patient from the Chad Republic, Africa. Eur J Clin Microbiol Infect Dis. 2009;28: 697–699. doi:10.1007/s10096-008-0668-2

6. Derrien JP, Vedy J, Monnier A. [African pulmonary histoplasmosis caused by Histoplasma duboisii (1st case in Chad)]. Bull Soc Med Afr Noire Lang Fr. 1978;23: 210–3. Available: http://www.ncbi.nlm.nih.gov/pubmed/719827

7. Ahogo KC, Sangaré A, Gbery IP, Ecra E, Kaloga M, Kassi K, et al. [Cutaneous histoplasmosis due to Histoplasma capsulatum variety duboisii in an immune competent child. About one case in Abidjan, Côte d’Ivoire]. Bull Soc Pathol Exot. Service de dermatologie du Centre hospitalier universitaire de Treichville, BP V 3 Abidjan, Côte d’Ivoire. ahogoceleste@yahoo.fr; 2009;102: 147–9. Available: http://europepmc.org/abstract/MED/19739407

8. Koffi N, Boka JB, Anzouan-Kacou JB, Aka-Danguy E. [African histoplasmosis with ganglionic localisation. Apropos of 1 case in an HIV negative patient]. Bull Soc Pathol Exot. 1997;90: 182–3. Available: http://www.ncbi.nlm.nih.gov/pubmed/9410255

9. Sangaré A, Yoboué P, Ahogo C, Ecra E, Kaloga M, Gbery I, et al. À propos d ’ un cas à Abidjan , Côte d ’ Ivoire . 2008; 5–7.

10. Bankolé Sanni R, Denoulet C, Coulibaly B, Nandiolo R, Kassi E, Honde M, et al. [Apropos of 1 Ivoirian case of osseus and cutaneous histoplasmosis by Histoplasma capsulatum var. duboisii]. Bull Soc Pathol Exot. 1998;91: 151–3. Available: http://www.ncbi.nlm.nih.gov/pubmed/9642470

11. Mambie A, Pasquet A, Melliez H, Bonne S, Blanc A-L, Patoz P, et al. A case of immune reconstitution inflammatory syndrome related to a disseminated histoplasmosis in an HIV-1 infected patient. AIDS. 2013;27: 2170–2. doi:10.1097/01.aids.0000432448.53110.e3

12. Orio J, Drouchet E, Gaillard C, N’da K, Pontich G. [Existence of capsulatum form histoplasmosis on the Ivory Coast. Description of first autochthonous human case with isolation of the strain]. Bull Soc Pathol Exot Filiales. 1968;61: 162–9. Available: http://www.ncbi.nlm.nih.gov/pubmed/4303035

13. Valmary J, Bauduceau B, Lartisien D, Flechaire A, Debord T, Daly JP, et al. [Disseminated Histoplasma duboisii histoplasmosis in a female inhabitant of the Ivory Coast]. Med Trop (Mars). 44: 369–73. Available: http://www.ncbi.nlm.nih.gov/pubmed/6097790

14. Imbert P, Poizot-Martin I, Lacour JP, Marty P, Dhiver C, Martet G. [Disseminated Histoplasma capsulatum histoplasmosis in African AIDS patients (3 cases)]. Med Trop (Mars). 1995;55: 151–3. Available: http://www.ncbi.nlm.nih.gov/pubmed/7564997

15. Inojosa W, Rossi MC, Laurino L, Giobbia M, Fuser R, Carniato A, et al. Progressive disseminated histoplasmosis among human immunodeficiency virus-infected patients from West-Africa: report of four imported cases in Italy. Infez Med. 2011;19: 49–55. Available: http://www.ncbi.nlm.nih.gov/pubmed/21471748

16. Oddo D, Etchart M, Thompson L. Histoplasmosis duboisii (African histoplasmosis). An African case reported from Chile with ultrastructural study. Pathol Res Pract. 1990;186: 514–7; discussion 518. doi:10.1016/S0344-0338(11)80473-5

17. Dietrich PY, Bille J, Fontolliet C, Regamey C. [Disseminated histoplasmosis due to Histoplasma capsulatum in a patient with acquired immunodeficiency syndrome (AIDS)]. Schweiz Med Wochenschr. 1987;117: 1289–96. Available: http://www.ncbi.nlm.nih.gov/pubmed/3313689

18. Lamey B, Parisien G. [Disseminated form of histoplasmosis caused by Histoplasma capsulatum in a Zairian child]. Med Trop (Mars). 42: 557–9. Available: http://www.ncbi.nlm.nih.gov/pubmed/6296597

19. Geffray L, Veyssier P, Cevallos R, Beaud B, Mayolle J, Nogier C, et al. [African histoplasmosis: clinical and therapeutic aspects, relation to AIDS. Apropos of 4 cases, including a case with HIV-1-HTLV-1 co-infection]. Ann Med Interne (Paris). 1994;145: 424–8. Available: http://www.ncbi.nlm.nih.gov/pubmed/7864504

20. Colebunders R, van den Abbeele K, Hauben E, Verstraeten T, Heremans T, van den Ende J, et al. Histoplasma capsulatum infection in three AIDS patients living in Africa. Scand J Infect Dis. 1995;27: 89–91. Available: http://www.ncbi.nlm.nih.gov/pubmed/7784825

21. Débat Zoguéreh D, Bigel M-L, Billy C, Perronne V, Richardin F, Granier F. [Disseminated histoplasmosis revealed by peripheral blood smear in an African immigrant with AIDS]. Med Mal Infect. 2008;38: 228–30. doi:10.1016/j.medmal.2007.10.009

22. Scarlata F, Imburgia C, Trizzino M, Titone L. [Leprosy-like cutaneous presentation of Histoplasma capsulatum infection in an African HIV+ patient]. Infez Med. 2012;20: 211–3. Available: http://www.ncbi.nlm.nih.gov/pubmed/22992563

23. Buhk T, Stellbrink H-J, Albrecht H, Sobottka I. [Severe colitis due to Histoplasma capsulatum in an AIDS patient]. Z Gastroenterol. 2006;44: 603–7. doi:10.1055/s-2006-926645

24. de Vries PJ, Koolen MGJ, Mulder MMS, Kortbeek LM. Acute pulmonary histoplasmosis from Ghana. Travel Med Infect Dis. 2006;4: 286–9. doi:10.1016/j.tmaid.2005.10.004

25. Rivasi F, Casali B, Nanetti A, Collina G, Mazzoni A. Histoplasma capsulatum var. capsulatum occurring in an HIV-positive Ghanaian immigrant to Italy. Identification of H. capsulatum DNA by PCR from paraffin sample. APMIS. 2001;109: 721–5. Available: http://www.ncbi.nlm.nih.gov/pubmed/11900050

26. de Hoog SH, Blok WL, van Ogtrop ML, van den Berk GEL. An unusual peripheral blood smear. Neth J Med. 2014;72: 332, 336. Available: http://www.ncbi.nlm.nih.gov/pubmed/25319861

27. Navarro M, Segura F, Font B, Espasa M, Taján J, Sala M, et al. Disseminated Infection by Mycobacterium sherrisii and Histoplasma capsulatum in an African HIV-Infected Patient. Am J Trop Med Hyg. 2013;88: 914–917. doi:10.4269/ajtmh.12-0572

28. Valero C, Gago S, Monteiro MC, Buitrago MJ. African histoplasmosis : new clinical and microbiological insights. 2017; 1–9. doi:10.1093/mmy/myx020

29. Murata M, Furusyo N, Otaguro S, Nabeshima S, Ariyama I, Hayashi J. HIV infection with concomitant cerebral toxoplasmosis and disseminated histoplasmosis in a 45-year-old man. J Infect Chemother. 2007;13: 51–5. doi:10.1007/s10156-006-0486-3

30. Kourda N, Mlika M, Zidi YSH, Zermani R, Ben Jilani S. [Cecal perforation in a kidney transplant patient: disseminated histoplasmosis]. Med Trop (Mars). 2010;70: 533–6. Available: http://www.ncbi.nlm.nih.gov/pubmed/21520661

31. Diongue K, Diallo MA, Badiane AS, Seck MC, Ndiaye M, Ndoye NW, et al. [Nondermatophytic and noncandidal fungi isolated in Le Dantec University hospital of Dakar in 2014: Epidemiological, clinical and mycological study]. J Mycol Med. 2015;25: 181–90. doi:10.1016/j.mycmed.2015.05.003

32. Ndiaye D, Diallo M, Sene PD, Ndiaye M, Ndir O. [Disseminated histoplasmosis due to Histoplasma capsulatum var. duboisii in Senegal. A case in HIV-infected patient]. J Mycol Med. 2011;21: 60–4. doi:10.1016/j.mycmed.2010.12.004

33. Dieng T, Massaly A, Sow D, Vellaissamy S, Sylla K, Tine RC, et al. Amplification of blood smear DNA to confirm disseminated histoplasmosis. Infection. 2017; doi:10.1007/s15010-017-0989-0

34. BASSET A, BASSET M, HOCQUET P. [Cutaneous forms of African histoplasmosis]. Bull Soc Fr Dermatol Syphiligr. 70: 61–4. Available: http://www.ncbi.nlm.nih.gov/pubmed/13969883

35. Privat Y, Faye I, Bellossi A. [Clinical polymorphism of histoplasmosis (presentation of a case with the aspect of leishmaniasis)]. Bull Soc Fr Dermatol Syphiligr. 1968;75: 475–6. Available: http://www.ncbi.nlm.nih.gov/pubmed/5702621

36. Diadie S, Diatta B, Ndiaye M, Gaye M, Sow D, Ndiaye MT, et al. [Multifocal histoplasmosis due to Histoplasma capsulatum var. duboisii in a 22 year-old Senegalese patient without proven immunodepression]. J Mycol Med. 2016;26: 265–70. doi:10.1016/j.mycmed.2016.03.004

37. Minta DK, Dembélé M, Lorre G, Diallo DA, Traoré HA, Chabasse D. [African histoplasmosis (Histoplasma capsulatum var. duboisii): a case report from Mali]. Sante. 15: 195–9. Available: http://www.ncbi.nlm.nih.gov/pubmed/16207583

38. Imperato PJ, Bridge MF. Histoplasma duboisii in the Republic of Mali. Trop Geogr Med. 1971;23: 79–83. Available: http://www.ncbi.nlm.nih.gov/pubmed/5573583

39. Llibre JM, Salvador A, Casanova J, Gallés C. [Persistent fever, retroperitoneal adenopathies, pancytopenia, and hepatosplenomegaly in an African immigrant with HIV-1 infection]. Enferm Infecc Microbiol Clin. 1996;14: 499–500. Available: http://www.ncbi.nlm.nih.gov/pubmed/9011209

40. Minta DK, Sylla M, Traoré AM, Soukho-Kaya A, Coulibaly I, Diallo K, et al. [Malian first observation of disseminated African histoplasmosis with predominant bone localizations in an HIV-negative child in Bamako (Mali). Review of the literature]. J Mycol Med. 2014;24: 152–7. doi:10.1016/j.mycmed.2013.08.001

41. Bayo S, Duflo B. [4 new cases of African Histoplasma duboisii histoplasmosis observed in Mali]. Dakar Med. 1979;24: 293–8. Available: http://www.ncbi.nlm.nih.gov/pubmed/546614

42. Tsiodras S, Drogari-Apiranthitou M, Pilichos K, Leventakos K, Kelesidis T, Buitrago MJ, et al. An unusual cutaneous tumor: African histoplasmosis following mudbaths: case report and review. Am J Trop Med Hyg. 2012;86: 261–3. doi:10.4269/ajtmh.2012.11-0557

43. N’Golet A, N’Gouoni BG, Moukassa D, Nkoua-Mbon JB. Maxillary African histoplasmosis: unusual diagnostic problems of an unusual presentation. Pathol Res Pract. 2005;200: 841–4. doi:10.1016/j.prp.2004.07.005

44. Arendt V, Coremans-Pelseneer J, Gottlob R, Bril T, Bujan-Boza W, Fondu P. African histoplasmosis in a Belgian AIDS patient. Mycoses. 34: 59–61. Available: http://www.ncbi.nlm.nih.gov/pubmed/1922190

45. Renoirte R, Michaux JL, Gatti F, Vanbreuseghem R, Bastin JP, Drexler L, et al. [New cases of African histoplasmosis and cryptococcosis observed in the Republic of the Congo]. Bull Acad R Med Belg. 1967;7: 465–527. Available: http://www.ncbi.nlm.nih.gov/pubmed/5607675

46. Therby A, Polotzanu O, Khau D, Monnier S, Greder Belan A, Eloy O. [Aspergillus galactomannan assay for the management of histoplasmosis due to Histoplasma capsulatum var. duboisii in HIV-infected patients: education from a clinical case]. J Mycol Med. 2014;24: 166–70. doi:10.1016/j.mycmed.2014.01.002

47. Bilkenroth U, Holzhausen HJ. [Disseminated infection by Histoplasma capsulatum with AIDS]. Pathologe. 2001;22: 270–5. Available: http://www.ncbi.nlm.nih.gov/pubmed/11490941

48. Ondzotto G, Ibara JR, Mowondabeka P, Galiba J. [Cervico-facial and ENT symptoms due to HIV infection in tropical area. About 253 Congolese cases]. Bull Soc Pathol Exot. 2004;97: 59–63. Available: http://www.ncbi.nlm.nih.gov/pubmed/15104161

49. Ngatse-Oko A, Péko JF, Ntsiba H, Ngolet A, Kokolo J, Ondzoto M, et al. [Pathological fracture revealing an osseous histoplasmosis. A case report on a 60-year patient]. Bull Soc Pathol Exot. 2006;99: 227–9. Available: http://www.ncbi.nlm.nih.gov/pubmed/17111967

50. RENDIRTE R, MICHAUX JL, GATTI F, VANBREUSEGHEM R. New African histoplasmosis and cryptococcosis cases in the democratic Congo republic. Bull Acad r, Med Belg. 1967;7: 465–526.

51. VANDEPITTE J, LAMOTE J, THYS A, VANBREUSE-GHEM R. Second Congo case of Histoplasmosis caused by Histoplasma duboisii Vanbreuseghem, 1952. Ann Soc Belg Med Trop (1920). Bruxelles: Societe Belge de Medecine Tropicale; 1957;37: 515–527.

52. RESSELER JJC, FARRIOR HL, VANBREUSEGHEM R. Two New Cases from the Congo of Histoplasmosis caused by Histoplasma duboisii. Ann Soc Belg Med Trop (1920). Bruxelles: Societe Belge de Medecine Tropicale; 1962;42: 801–814.

53. Loulergue P, Bastides F, Baudouin V, Chandenier J, Mariani-Kurkdjian P, Dupont B, et al. Literature review and case histories of Histoplasma capsulatum var. duboisii infections in HIV-infected patients. Emerg Infect Dis. 2007;13: 1647–1652. doi:10.3201/eid1311.070665

54. Borges-Costa J, Marques T, Soares-Almeida L, Sacramento-Marques M. Progressive disseminated histoplasmosis as a presentation of AIDS in a patient from the Congo: the role of skin biopsy. Trop Doct. 2011;41: 251–252. doi:10.1258/td.2011.110140

55. Chandenier J, Goma D, Moyen G, Samba-Lefèbvre MC, Nzingoula S, Obengui, et al. [African histoplasmosis due to Histoplasma capsulatum var. duboisii: relationship with AIDS in recent Congolese cases]. Sante. 5: 227–34. Available: http://www.ncbi.nlm.nih.gov/pubmed/7582643

56. Carme B, Ngaporo AI, Ngolet A, Ibara JR, Ebikili B. Disseminated African histoplasmosis in a Congolese patient with AIDS. J Med Vet Mycol. Taylor & Francis; 1992;30: 245–248. doi:10.1080/02681219280000301

57. Carme B, Ngolet A, Ebikili B, Ngaporo AI. Is African histoplasmosis an opportunistic fungal infection in AIDS? Trans R Soc Trop Med Hyg. 1990;84: 293. doi:10.1016/0035-9203(90)90292-M

58. Mabiala Babela JR, Mboutol Mandavo C, Nika Evrard R, Ossibi Ibara B, Lamah L, Ollandzobo Ikobo LC, et al. [African histoplamosis. A report of three pediatric cases]. J Mycol Med. 2017;27: 133–138. doi:10.1016/j.mycmed.2017.01.013

59. Bashiri SA, Shahmirzadi MRR, Abro AH. A case of disseminated histoplasmosis which was misdiagnosed as squamous cell carcinoma of skin and pulmonary tuberculosis;case report, Rashid hospital,2013. Gulf Congress of Clinical Microbiology & Infectious Disease. Dubai, United Arab Emirates; 2016. p. PP05.

60. Daramola JO, Ajagbe HA, Abioye AA, Ogunba EO. Maxillary african histoplasmosis mimicking malignant jaw tumour. Br J Oral Surg. 1979;16: 241–247. doi:10.1016/0007-117X(79)90030-1

61. Ubesie A, Okafo O, Ibeziako N, Onukwuli V, Mbanefo N, Uzoigwe J, et al. Disseminated Histoplasmosis in a 13-year-old girl: A case report. Afr Health Sci. 2013;13: 518–21. doi:10.4314/ahs.v13i2.45

62. Akpuaka FC, Gugnani HC, Iregbulam LM. African histoplasmosis: report of two patients treated with amphotericin B and ketoconazole. Mycoses. Blackwell Publishing Ltd; 1998;41: 363–364. doi:10.1111/j.1439-0507.1998.tb00354.x

63. KA Adeniji AA. Peripheral lymphadenopathy in Nigeria. Afr J Med Med Sci. 2000;29: 233–237.

64. Jacyk WK, Lawande R V, Tulpule SS. Deep Mycoses in West Africa: A Report of 13 Cases and Review of the Nigerian Literature. J Natl Med Assoc. 1981;73: 251–259. Available: http://www.ncbi.nlm.nih.gov/pmc/articles/PMC2609798/

65. LUCAS AO. CUTANEOUS MANIFESTATIONS OF AFRICAN HISTOPLASMOSIS ADETOKUNBO O. LUCAS. Br J Dermatol. Blackwell Publishing Ltd; 1970;82: 435–447. doi:10.1111/j.1365-2133.1970.tb02203.x

66. Adekunle OO, Sudhakaran P, Timeyin ED. African histoplasmosis of the jejunum. Report of a case. J Trop Med Hyg. 1978;81: 88–90. Available: http://www.ncbi.nlm.nih.gov/pubmed/660708

67. Onuigbo WI, Gugnani HC. Deep mycoses prevalent in the Igbos of Nigeria. Int J Dermatol. 15: 432–7. Available: http://www.ncbi.nlm.nih.gov/pubmed/1279071

68. Solanke TF, Akinyemi OO, Clark BM. A case of histoplasmosis in a Nigerian. J Trop Med Hyg. 1969;72: 101–4. Available: http://www.ncbi.nlm.nih.gov/pubmed/5769716

69. Akinosi JO. African histoplasmosis presenting as a dental problem. Br J Oral Surg. 1970;8: 58–63. doi:10.1016/S0007-117X(70)80069-5

70. Oguachuba HN, Gugnani HC. African histoplasmosis manifesting as a cutaneous tumour treated with econazole. J Trop Med Hyg. 1982;85: 259–63. Available: http://www.ncbi.nlm.nih.gov/pubmed/7154150

71. Olasoji HO, Pindiga UH, Adeosun OO. African oral histoplasmosis mimicking lip carcinoma: case report. East Afr Med J. 1999;76: 475–6. Available: http://www.ncbi.nlm.nih.gov/pubmed/10520359

72. Akinyoola AL, Onayemi O, Famurewa OC. African histoplasmosis - masquerading as a malignant bone tumour. Trop Doct. 2006;36: 250–251. doi:10.1258/004947506778604922

73. Onwuasoigwe O, Gugnani HC. African histoplasmosis: osteomyelitis of the radius. Mycoses. 41: 105–7. Available: http://www.ncbi.nlm.nih.gov/pubmed/9670760

74. Khalil M, Iwatt AR, Gugnani HC. African histoplasmosis masquerading as carcinoma of the colon. Dis Colon Rectum. 1989;32: 518–520. doi:10.1007/BF02554509

75. Khalil M, Ekanem IO, Gugnani HC, Attah EB. Some deep mycoses diagnosed by histopathology in South Eastern Nigeria. Rev Iberoam Micol. 1999;16: 221–4. Available: http://www.ncbi.nlm.nih.gov/pubmed/18473552

76. Adekeye EO, Edwards MB, Williams HK. Mandibular African histoplasmosis: Imitation of neoplasia or giant-cell granuloma? Oral Surgery, Oral Med Oral Pathol. 1988;65: 81–84. doi:10.1016/0030-4220(88)90197-1

77. Egere JU, Gugnani HC, Okoro AN, Suseelan A V. African histoplasmosis in Eastern Nigeria: report of two culturally proven cases treated with septrin and amphotericin B. J Trop Med Hyg. 1978;81: 225–9. Available: http://www.ncbi.nlm.nih.gov/pubmed/731765

78. Khalil MA, Hassan AW, Gugnani HC. African histoplasmosis: report of four cases from northeastern Nigeria. Mycoses. Blackwell Publishing Ltd; 1998;41: 293–295. doi:10.1111/j.1439-0507.1998.tb00341.x

79. WILLIAMS AO, LAWSON EA, Lucas AO. African histoplasmosis due to Histoplasma duboisii. Arch Pathol. 1971;92: 306–18. Available: http://www.ncbi.nlm.nih.gov/pubmed/5165016

80. Mace MC. Oral African histoplasmosis resembling Burkitt’s lymphoma. Oral Surgery, Oral Med Oral Pathol. 1978;46: 407–412. doi:10.1016/0030-4220(78)90406-1

81. Shoroye A, Oyedeji GA. African histoplasmosis presenting as a facial tumour in a child. Ann Trop Paediatr. 1982;2: 147–149. doi:10.1080/02724936.1982.11748248

82. Uloko A, Maiyaki M, Nagoda M, Babashani M. Histoplasmosis: An elusive re-emerging chest infection. Niger J Clin Pract. Medknow Publications and Media Pvt. Ltd.; 2012;15: 235. doi:10.4103/1119-3077.97336

83. Seriki O, Aderele WI, Johnson A, Smith JA. Disseminated histoplasmosis due to histoplasma capsulatum in two Nigerian children. J Trop Med Hyg. 1975;78: 248–55. Available: http://www.ncbi.nlm.nih.gov/pubmed/1223326

84. Asamoa EA, Ayanlere AO, Olaitan AA, Adekeye EO. Paediatric tumours of the jaws in northern Nigeria. J Cranio-Maxillofacial Surg. 1990;18: 130–135. doi:10.1016/S1010-5182(05)80330-0

85. Samaila MO, Abdullahi K. Cutaneous manifestations of deep mycosis: An experience in a tropical pathology laboratory. Indian J Dermatol. Wolters Kluwer -- Medknow Publications; 2011;56: 282. doi:10.4103/0019-5154.82481

86. Seleye-Fubara D, Etebu E, Bob-Yellowe E. Granulomatous osteomyelitis: *A review of 13 cases in Port Harcourt, Nigeria*. Sahel Med J. 2011;14: 11–15. Available: http://www.smjonline.org/article.asp?issn=1118-8561

87. CLARK BM, GREENWOOD BM. Pulmonary lesions in African histoplasmosis. J Trop Med Hyg. 1968;71: 4–10.

88. Bansal RK, Suseelan AV, Gugnani HC. Orbital cyst due to Histoplasma duboisii. Br J Ophthalmol. 1977;61: 70–71.

89. Olurin O, Lucas AO, Oyediran ABO. Orbital Histoplasmosis Due to Histoplasma Duboisii. Am J Ophthalmol. 1969;68: 14–18. doi:10.1016/0002-9394(69)94929-0

90. Ige AO, Nwosu SO, Odesanmi WO. African histoplasmosis (Duboisii) of the skull with neurological complication--a case report and review of literature. Afr J Med Med Sci. 1992;21: 19–21. Available: http://www.ncbi.nlm.nih.gov/pubmed/1308076

91. Bayés B, Romeu J, Vaquero M, Ribera M, Navarro JT, Rosell A, et al. [Disseminated histoplasmosis and AIDS. Report of 4 cases]. Med Clin (Barc). 1996;106: 700–3. Available: http://www.ncbi.nlm.nih.gov/pubmed/8801374

92. Eichmann A, Schär G. [African histoplasmosis in a patient with HIV-2 infection]. Schweiz Med Wochenschr. 1996;126: 765–9. Available: http://www.ncbi.nlm.nih.gov/pubmed/8693301

93. Pitche P, Dossim A, Mijiyawa M, Napo-Koura G, Tchangaï-Walla K. [Multiple bone lesions of a type of disseminated African histoplasmosis in a Togolese immunocompetent child]. Rev Chir Orthop Reparatrice Appar Mot. 1995;81: 745–48. Available: http://www.ncbi.nlm.nih.gov/pubmed/8761658

94. Fritzsche C, Loebermann M, Aepinus C, Bolz M, Barten M, Reisinger ECC, et al. Vaginal Ulceration and Local Lymphadenopathy in an African Immigrant. Clin Infect Dis. Oxford University Press; 2009;48. Available: https://academic.oup.com/cid/article-lookup/doi/10.1086/596473

95. Simon F, Chouc PY, Hervé V, Branquet D, Jeandel P. [Bone and joint sites of African histoplasmosis (Histoplasma duboisii). Apropos of a case and review of the literature]. Rev Rhum Ed Fr. Service de Médecine, Hôpital Régional de Bambari, République Centrafricaine (1), Marseille Armées.; 1994;61: 829–838. Available: http://www.ncbi.nlm.nih.gov/pubmed/7858578

96. Coulanges P, Raveloarison G, Ravisse P. Existence of histoplasmosis with Histoplasma duboisii outside Continental Africa (the first case from Madagascar). Bull Soc Pathol Exot Filiales. 1982;75: 400–403.

97. Coulanges P. [Large-form histoplasmosis (H. duboisii) in Madagascar (apropos of 3 cases)]. Arch Inst Pasteur Madagascar. 1989;56: 169–74. Available: http://www.ncbi.nlm.nih.gov/pubmed/2633706

98. Chauvet E, Carreiro M, Berry A, Tohfe M, Ollier S, Sailler L, et al. Une histoplasmose du plancher buccal 34 ans après un retour d’Afrique. La Rev Médecine Interne. 2003;24: 195–197. doi:10.1016/S0248-8663(02)00807-X

99. Molina L, Garau M, García J, Odriozola M, del Palacio A. [Three cases of imported histoplasmosis in our hospital]. Rev Iberoam Micol. 2002;19: 204–7. Available: http://www.ncbi.nlm.nih.gov/pubmed/12825983

100. Torres-Rodríguez JM, Segura-Roca G, Coll J. [Histoplasmosis 45 years after infection in an immunocompetent man]. Rev Iberoam Micol. 2009;26: 244–6. doi:10.1016/j.riam.2009.03.004

101. MÁRTON K, MÁRTON-ECSI E, LAMA A, DIALIO AO. The first case of osseo-cutaneous histoplasmosis seen in Guinea. Int J Dermatol. 1974;13: 190–196. doi:10.1111/j.1365-4362.1974.tb01792.x

102. Nethercott JR, Schachter RK, Givan KF, Ryder DE. Histoplasmosis Due to Histoplasma capsulatum var duboisii in a Canadian Immigrant. Arch Dermatol. American Medical Association; 1978;114: 595. doi:10.1001/archderm.1978.01640160073023

103. Gumaa SA, Ahmed MA, Hassan MEA, Hassan AME. A case of African histoplasmosis from Sudan. Trans R Soc Trop Med Hyg. 1988;82: 503–505. doi:10.1016/0035-9203(88)90178-2

104. Medical Association of East Africa. M V., British Medical Association. T, Kenya Medical Association. H. The East African medical journal : the organ of the Medical Association of East Africa. [Internet]. East African Medical Journal. Medical Association of East Africa; 1978. Available: https://www.cabdirect.org/cabdirect/abstract/19792901725

105. Pamnani R, Rajab J, Githang’a J, Kasmani R. Disseminated histoplasmosis diagnosed on bone marrow aspirate cytology: Report of four cases. East Afr Med J. Kenya Medical Association; 2010;86: 102–105. doi:10.4314/eamj.v86i12.62918

106. McCLATCHIE S, KUNGU A. Intestinal histoplasmosis in a Kenya African. East Afr Med J. 1969;46: 166–169.

107. Wasunna KM, Chunge CN, Gachihi G, Chulay J, Anabwani G, Riyat MS. Disseminated histoplasmosis in a Kenyan African child: a case report. East Afr Med J. 1985;62: 285–9. Available: http://www.ncbi.nlm.nih.gov/pubmed/4042938

108. Amayo EO, Riyat MS, Okelo GB, Adam AM, Toroitich K. Disseminated histoplasmosis in a patient with acquired immunodeficiency syndrome (AIDS): a case report. East Afr Med J. 1993;70: 61–2. Available: http://www.ncbi.nlm.nih.gov/pubmed/8513733

109. DAVIES P. A Fatal Case of Histoplasmosis contracted in Kenya. East Afr Med J. 1957;34: 555–557.

110. SHAH M V, OGADA T, NSANZUMUHIRE H. Case report of Histoplasma capsulatum pulmonary histoplasmosis in a Kenyan African. East Afr Med J. 1978;55: 438–441.

111. Leibowitz MC, Berson SD, Martin PM. Disseminated histoplasmosis associated with disseminated tuberculosis: a case report. S Afr Med J. 1977;51: 315–7. Available: http://www.ncbi.nlm.nih.gov/pubmed/847553

112. K Ramdial P, Mosam A, Dlova NC, B Satar N, Aboobaker J, Singh SM. Disseminated cutaneous histoplasmosis in patients infected with human immunodeficiency virus. J Cutan Pathol. 2002;29: 215–25. Available: http://www.ncbi.nlm.nih.gov/pubmed/12028154

113. Craven SA, Benatar SR. Histoplasmosis in the Cape Province. A report of the second known outbreak. S Afr Med J. 1979;55: 89–92. Available: http://www.ncbi.nlm.nih.gov/pubmed/570729

114. Hodgson T, Rachanis C. Oral fungal and bacterial infections in HIV-infected individuals: an overview in Africa. Oral Dis. 2002;8: 80–87. doi:10.1034/j.1601-0825.2002.00017.x

115. Sen D, Birns J, Rahman A. Articular presentation of disseminated histoplasmosis. Clin Rheumatol. 2007;26: 823–824. doi:10.1007/s10067-006-0331-1

116. Mosam A, Moodley V, Ramdial PK, Sathar N, Aboobaker J, Singh S. Persistent pyrexia and plaques: a perplexing puzzle. Lancet (London, England). Elsevier; 2006;368: 551. doi:10.1016/S0140-6736(06)69166-6

117. Murphy RA, Gounder L, Manzini TC, Ramdial PK, Castilla C, Moosa M-YS. Challenges in the management of disseminated progressive histoplasmosis in human immunodeficiency virus-infected patients in resource-limited settings. Open forum Infect Dis. 2015;2: ofv025. doi:10.1093/ofid/ofv025

118. KLUGMAN HB, LURIE HI. Systemic histoplasmosis in South Africa. A review of the previous cases and a report of an additional case--the first successfully treated. S Afr Med J. 1963;37: 29–31. Available: http://www.ncbi.nlm.nih.gov/pubmed/14033690

119. MURRAY JF, LURIE HI, KAYE J, KOMINS C, BOROK R, WAY M. Benign pulmonary histoplasmosis (cave disease) in South Africa. S Afr Med J. 1957;31: 245–53. Available: http://www.ncbi.nlm.nih.gov/pubmed/13421901

120. Dawood H. A Case of Immune Reconstitution Syndrome to Disseminated Histoplasmosis. J Int Assoc Physicians AIDS Care. SAGE PublicationsSage CA: Los Angeles, CA; 2011;10: 277–279. doi:10.1177/1545109711408316

121. Mackenjee MK, Coovadia HM. Histoplasmosis treated with a sulphonamide. A case report. S Afr Med J. 1976;50: 2015–6. Available: http://www.ncbi.nlm.nih.gov/pubmed/1006475

122. Scheepers A, Lemmer J. Disseminated histoplasmosis: aspects of oral diagnosis. J Dent Assoc S Afr. 1992;47: 441–3. Available: http://www.ncbi.nlm.nih.gov/pubmed/9511606

123. HAZELHURST JA, VISMER HF. Histoplasmosis presenting with unusual skin lesions in acquired immunodeficiency syndrome (AIDS). Br J Dermatol. 1985;113: 345–348. doi:10.1111/j.1365-2133.1985.tb02088.x

124. Rajah V, Essa A. Histoplasmosis of the oral cavity, oropharynx and larynx. J Laryngol Otol. 1993;107: 58–61. Available: http://www.ncbi.nlm.nih.gov/pubmed/8445319

125. Pillay T, Pillay DG, Bramdev A. DISSEMINATED HISTOPLASMOSIS IN A HUMAN IMMUNODEFICIENCY VIRUS-INFECTED AFRICAN CHILD. Pediatr Infect Dis J. 1997;16. Available: http://journals.lww.com/pidj/Fulltext/1997/04000/DISSEMINATED_HISTOPLASMOSIS_IN_A_HUMAN.19.aspx

126. Bank S, Trey C, Gans I, Marks IN, Groll A. Histoplasmosis of the small bowel with “giant” intestinal villi and secondary protein-losing enteropathy. Am J Med. 1965;39: 492–501. doi:10.1016/0002-9343(65)90216-0

127. White J, Khammissa R, Wood NH, Meyerov R, Lemmer J, Feller L. Oral histoplasmosis as the initial indication of HIV infection: a case report. SADJ. 2007;62: 452, 454–5. Available: http://www.ncbi.nlm.nih.gov/pubmed/18500107

128. Chihab W, Achergui A, Agoumi A, Chraïbi H, Hassam B, Mansouri F. [American histoplasmosis: a case with cutaneous presentation in Morocco]. Med Trop (Mars). 2003;63: 171–4. Available: http://www.ncbi.nlm.nih.gov/pubmed/12910657

129. Elansari R, Abada R, Rouadi S, Roubal M, Mahtar M. Histoplasma capsulatum sinusitis: Possible way of revelation to the disseminated form of histoplasmosis in HIV patients. Int J Surg Case Rep. 2016;24: 97–100. doi:10.1016/j.ijscr.2016.03.010

130. Tazi EM, Essadi I, Serraj K, Ichou M, Errihani H. Histoplasmose sacrée dix ans après un lymphome non hodgkinien du sacrum : à propos d’un cas. Cancer/Radiothérapie. 2009;13: 337–339. doi:10.1016/j.canrad.2009.03.004

131. BROWN KGE, MOLESWORTH BD, BOERRIGTER FGG, TOZER RA. Disseminated Histoplasmosis duboisii in Malawi. Partial response to sulphonamide/trimethoprim combination. East Afr Med J. 1974;51: 582–590.

132. Kalongolera L, Kamiza S, Bates J, Harrison WJ, van Oosterhout JJ. Histoplasmosis in a Malawian patient on ART. Malawi Med J. 2013;25: 93. Available: http://www.ncbi.nlm.nih.gov/pubmed/24358429

133. Sharma D, McKendry A, Nageshwaran S, Cartledge J. A case of oral ulceration and disseminated histoplasmosis in HIV infection. Int J STD AIDS. 2012;23: 522–3. doi:10.1258/ijsa.2011.011261

134. Dierckxsens H, Vanderick F, Vandepitte J, Ntabomvura V. [1st cases of histoplasmosis caused by Histoplasma capsulatum in Rwanda]. Ann Soc Belg Med Trop (1920). 1976;56: 1–10. Available: http://www.ncbi.nlm.nih.gov/pubmed/1015875

135. Jadin JB, Vanderick F, Mbonyingabo. First case of Histoplasma duboisi in Rwanda. Trans R Soc Trop Med Hyg. 1972;66: 14. Available: http://www.ncbi.nlm.nih.gov/pubmed/5048065

136. Régnier-Rosencher E, Dupont B, Jacobelli S, Paugam A, Carlotti A, Boitier F, et al. Late occurrence of Histoplasma duboisii cutaneous and pulmonary infection 18 years after exposure. J Mycol Med. 2014;24: 229–33. doi:10.1016/j.mycmed.2014.08.001

137. Abrucio Neto L, Takahashi MD, Salebian A, Cucé LC. African histoplasmosis. Report of the first case in Brazil and treatment with itraconazole. Rev Inst Med Trop Sao Paulo. 35: 295–9. Available: http://www.ncbi.nlm.nih.gov/pubmed/8278762

138. Gatti F, Renoirte R, Vanbreuseghem R. [African histoplasmosis and chromoblastomycosis in Angolians]. Ann Soc Belges Med Trop Parasitol Mycol. 1967;47: 249–56. Available: http://www.ncbi.nlm.nih.gov/pubmed/5619092

139. SOBRALFDA C, DA SILVA JN. [THE FIRST ANGOLESE CASE OF AFRICAN HISTOPLASMOSIS]. J Soc Cienc Med Lisb. 1963;127: 731–47. Available: http://www.ncbi.nlm.nih.gov/pubmed/14115001

140. FRETILLERE Y, PELOUX Y. First case of histoplasmosis observed on the French Somali Coast. Ulceration of the soft palate due to H. capsulatum. Med Trop. 1965;25: 341–343.

141. Rubach MP, Maro VP, Bartlett JA, Crump JA. Etiologies of illness among patients meeting Integrated Management of Adolescent and Adult Illness District Clinician Manual criteria for severe infections in northern Tanzania: Implications for empiric antimicrobial therapy. Am J Trop Med Hyg. 2015;92: 454–462. doi:10.4269/ajtmh.14-0496

142. Lofgren SM, Kirsch EJ, Maro VP, Morrissey AB, Msuya LJ, Kinabo GD, et al. Histoplasmosis among hospitalized febrile patients in northern Tanzania. Trans R Soc Trop Med Hyg. 2012;106: 504–507. doi:10.1016/j.trstmh.2012.05.009

143. Kabangila R, Semvua K, Rambau P, Jackson K, Mshana SE, Jaka H, et al. Pulmonary histoplasmosis presenting as chronic productive cough, fever, and massive unilateral consolidation in a 15-year-old immune-competent boy: a case report. J Med Case Rep. 2011;5: 374. doi:10.1186/1752-1947-5-374

144. Crump JA, Ramadhani HO, Morrissey AB, Msuya LJ, Yang L-Y, Chow S-C, et al. Invasive bacterial and fungal infections among hospitalized HIV-infected and HIV-uninfected children and infants in northern Tanzania. Trop Med Int Heal. 2011;16: 830–837. doi:10.1111/j.1365-3156.2011.02774.x

145. Crump JA, Ramadhani HO, Morrissey AB, Saganda W, Mwako MS, Yang L-Y, et al. Invasive Bacterial and Fungal Infections Among Hospitalized HIV-Infected and HIV-Uninfected Adults and Adolescents in Northern Tanzania. Clin Infect Dis. 2011;52: 341–348. doi:10.1093/cid/ciq103

146. Mignogna M, Fedele S, Lo Russo L, Ruoppo E, Lo Muzio L. A case of oral localized histoplasmosis in an immunocompetent patient. Eur J Clin Microbiol Infect Dis. 2001;20: 753–755. doi:10.1007/s100960100592

147. JOHNSTONE G. Histoplasmosis in Tanganyika (Tanzania). J Trop Med Hyg. 1965;68: 85–91.

148. DUCLOUX M, AGBANRIN E, VARANGO G. Histoplasmosis in Dahomey. Bull Soc Med Afr Noire Lang Fr. Societe Medicale d’Afrique Noire de Langue Francaise; 1970;15: 177–185.

149. Musoke F. Spinal African histoplasmosis simulating tuberculous spondylitis. Afr Heal Sci. 2001;1: 28–29.

150. Lanceley JL, Lunn HF, Wilson AMM. Histoplasmosis in an african child. J Pediatr. 1961;59: 756–764. doi:10.1016/S0022-3476(61)80017-6

151. MUGERWA JW. Histoplasma infection in Uganda. East Afr Med J. 1977;54: 227–232.

152. Raselli C, Reinhart WH, Fleisch F. [Histoplasmosis - an unusual African souvenir]. Dtsch Med Wochenschr. 2013;138: 313–6. doi:10.1055/s-0032-1332870

153. Warnakulasuriya KA, Harrison JD, Johnson NW, Edwards S, Taylor C, Pozniak AL. Localised oral histoplasmosis lesions associated with HIV infection. J Oral Pathol Med. 1997;26: 294–6. Available: http://www.ncbi.nlm.nih.gov/pubmed/9234191

154. Cottle LE, Gkrania-Klotsas E, Williams HJ, Brindle HE, Carmichael AJ, Fry G, et al. A multinational outbreak of histoplasmosis following a biology field trip in the Ugandan rainforest. J Travel Med. 20: 83–7. doi:10.1111/jtm.12012

155. MUTESASIRA L, TEMPLETON AC. Disseminated Histoplasmosis duboisii in Uganda. East Afr Med J. 1968;45: 687–693.

156. Nalwanga D, Henning L. If It Looks Like a Duck, Swims Like a Duck, and Quacks Like a Duck--Does It Have to Be a Duck? PLoS Negl Trop Dis. 2016;10: e0004430. doi:10.1371/journal.pntd.0004430

157. Kweyamba V, Apiyo M, Olika B, Kituuka O. Case Report A Case of a 4-Year-Old Boy with a Mesenteric Chylous Cyst Infected with Histoplasma capsulatum. 2016;2016: 3–7.

158. Kiggundu R, Nabeta HW, Okia R, Rhein J, Lukande R. Unmasking histoplasmosis immune reconstitution inflammatory syndrome in a patient recently started on antiretroviral therapy. Autops Case Reports. 2016;6: 27–33. doi:10.4322/acr.2016.048

159. Zida A, Niamba P, Barro-Traoré F, Korsaga-Somé N, Tapsoba P, Briegel J, et al. Disseminated histoplasmosis caused by Histoplasma capsulatum var. duboisii in a non-HIV patient in Burkina Faso: Case report. J Mycol Med. 2015;25: 159–62. doi:10.1016/j.mycmed.2015.03.002

160. PICQ JJ, RICOSSE JH, ALBERT JP, DROUHET E. 2 cases of African histoplasmosis in Upper Volta. Med Trop. 1968;28: 67–74.

161. André C, Badoual J, Kalifa G, Dubousset J. [African histoplasmosis. A case]. Arch Fr Pediatr. 41: 429–31. Available: http://www.ncbi.nlm.nih.gov/pubmed/6487046

162. Mandengue CE, Ngandjio A, Atangana PJA. Histoplasmosis in HIV-Infected Persons, Yaoundé, Cameroon. Emerg Infect Dis. 2015;21: 2094–2096. doi:10.3201/eid2111.150278

163. MENU P, REVIL H. [Case of pulmonary histoplasmosis found in Yaounde during a systematic examination]. Bull Soc Pathol Exot Filiales. 1955;48: 810–4. Available: http://www.ncbi.nlm.nih.gov/pubmed/13329742

164. Degos R, Badillet G, Sarrazin P, Hurtado S. [“Minor forms” of buccal histoplasmosis of Cameroon origin]. Bull Soc Fr Dermatol Syphiligr. 1970;77: 494–6. Available: http://www.ncbi.nlm.nih.gov/pubmed/5499477

165. Mandengue CE, Lindou J, Mandeng N, Takuefou B, Nouedoui C, Atangana P, et al. [Fatal miliary tuberculosis in an HIV-infected Cameroon woman: disseminated histoplasmosis due to Histoplasma capsulatum capsulatum]. Med Trop (Mars). 2011;71: 615–7. Available: http://www.ncbi.nlm.nih.gov/pubmed/22393632

166. Ebenye CM. A Case of Disseminated Histoplasmosis Detected in Peripheral Blood Smear Staining Revealing AIDS at Terminal Phase in a Female Patient from Cameroon. Case Rep Med. 2012;2012: 1–3. doi:10.1155/2012/215207

167. GAMET A, BROTTES H, CHATAIGNEAU P, BLOUZON J. Apropos the first cases of histoplasmosis observed in the Cameroons. Bull la Société Pathol Exot. Paris: Societe de Pathologie Exotique; 1962;55: 229–232.

168. Atangana PJA. Histoplasmose disséminée traitée par bolus de fl uconazole Disseminated histoplasmosis treated by boluses of fl uconazole. 2015; 110–111.
